# Noise-induced Hearing Loss among Patients Requiring Pure Tone Audiometry Evaluation in a Tertiary Care Centre: A Descriptive Cross-sectional Study

**DOI:** 10.31729/jnma.8008

**Published:** 2023-02-28

**Authors:** Sangita Shrestha, Biraj Baral, Aakriti Dawadi, Pema Sherpa, Deepak Regmi

**Affiliations:** 1 Department of Otorhinolaryngology, Kathmandu Medical College and Teaching Hospital, Sinamangal, Kathmandu, Nepal; 2 Kathmanau Medical College and Teaching Hospital, Sinamangal, Kathmandu, Nepal; 3 Maharajgunj Medical Campus, Maharajgunj, Kathmandu, Nepal

**Keywords:** *audiometry*, *noise-induced hearing loss*, *tinnitus*

## Abstract

**Introduction:**

Noise-induced hearing loss is a type of sensorineural hearing loss caused by longterm exposure to loud noise. This study provides insight into hearing loss problems the general population faces. The study aimed to find out the prevalence of noise-induced hearing loss among patients needing pure tone audiometry evaluation in a tertiary care centre.

**Methods:**

A descriptive cross-sectional study was conducted from 1 January 2021 to 30 July 2021 among patients requiring pure tone audiometry evaluation in the outpatient Department of Otorhinolaryngology in a tertiary care centre. The study was conducted after ethical approval from Institutional Review Committee (Reference number: 2812202001). Pure tone audiometry was used to diagnose noise-induced hearing loss. Convenience sampling was done. Point estimate and 95% Confidence Interval were calculated.

**Results:**

Out of 690 patients, 14 (2.02%) (0.97-3.06, 95% Confidence Interval) were diagnosed with noise-induced hearing loss.

**Conclusions:**

The prevalence of noise-induced hearing loss among patients requiring pure tone audiometry evaluation was similar to other studies conducted in similar settings.

## INTRODUCTION

Noise-induced hearing loss (NIHL) is a sensory neural type of hearing loss where chronic exposure to less intense sound damages cochlear hair cells and nerves.^1^ It has affected people of every age and every occupation in every part of the world. In the long run, NIHL increases morbidity and lowers the quality of life of those affected.

NIHL is one of the most common preventable causes of sensorineural hearing loss.^2^ Henceforth, understanding the prevalence of noise-induced hearing loss in our hospital scenario is imperative in providing medical professionals with the information to tackle these cases better.

This study aimed to find out the prevalence of NIHL among patients requiring pure tone audiometry (PTA) evaluation in a tertiary care centre.

## METHODS

A descriptive cross-sectional study was conducted in the Department of Otorhinolaryngology of Kathmandu Medical College and Teaching Hospital, Kathmandu, Nepal among patients requiring PTA evaluation from 1 January 2021 to 30 July 2021. All the audiogram reports of the patients reported at this time frame were evaluated for evidence of NIHL. Ethical approval was obtained from the Institutional Review Committee (Reference number: 2812202001). The audiometry was performed by a technologist trained in audiometric techniques. All ages of patients who underwent audiometrie assessment during the study period were included in the study. Convenience sampling was used.


$$\begin{array}{ll} \text{n} & ={\text{Z}}^{2}\times \frac{\text{p}\times \text{q}}{{\text{e}}^{\text{2}}}\\ & =1.96^{2}\times \frac{0.0182\times 0.9818}{0.01^{2}}\\ & =687 \end{array}$$

Where,

Z= 1.96 at 95% Confidence Interval (CI)
p = prevalence of noise-induced hearing loss taken from previous study as 1.82%^3^
q = 1-p
e = error of margin, 1%

The calculated sample size was 687. However, 690 samples were included in the study.

The diagnosis for noise-induced hearing loss is based on finding an audiometric notch between 3 kHz to 6 kHz, alongside a history of noise exposure and symptoms of muffled hearing loss, vertigo, and tinnitus.^4,5^ This method for diagnosis is widely used by different authors in kinds of literature and in clinical practice. While 4 kHz is the classic frequency affected, the notch can be noted elsewhere because of the noise frequency range in which cochlear damage occurs.6 Audiometric dip seen only on 4 kHz is thus referred to as a narrow dip (V type) otherwise it is said to be a wide dip (U type).^7^ As per study criteria, audiograms having simultaneous conductive hearing loss i.e. Air-Bone gap >10 dB were excluded for NIHL prevalence. The severity of NIHL was graded according to WHO standards after taking the average of the 4 frequencies i.e. 500 Hz, 1000 Hz, 2000 Hz, and 4000 Hz in the right and left ears separately.^8,9^ Patients with the NIHL result in even one ear were considered for diagnosis and included in prevalence.

Data were entered in Microsoft Excel 2016 and analyzed using IBM SPSS Statistics version 22.0. Point estimate and 95% CI were calculated.

## RESULTS

Among 690 participants, the prevalence of NIHL was 14 (2.02%) (0.97-3.06, 95% CI). The mean age of NIHL-affected individuals was 36.36±10.20 years. A total of 7 (50%) complained only of muffled hearing loss, 5 (35.71%) had muffled hearing loss with tinnitus, 1 (7.14%) had tinnitus while only 1 (7.14%) had all of these complaints. There were 5 (64.28%) females and 9 (35.71%) males. Most of the patients had both ears affected and only a few had unilateral NIHL. There were about 11 (78.57%) cases of binaural noise-induced hearing loss and 3 (21.40%) cases of unilateral hearing loss. In terms of unilateral involvement, 2 (14.28%) and 1 (7.14%) were seen only in the left and right ears, respectively.

The severity of the hearing loss is graded according to WHO standards. A total of 6 (42.85%) and 8 (57.14%) cases were in the mild range of hearing loss in the left and right ears respectively (Table 1).

**Table 1:** Distribution of WHO grading for severity of hearing loss (n= 14).

Severity (dB)	Left ear n (%)	Right ear n (%)
Normal (0-25)	7 (50)	6 (42.90)
Mild (26-40)	6 (42.90)	8 (57.10)
Moderate (41-60)	1 (7.10)	-

In the left ear 5 (35.71%) had a narrow dip and 7 (50%) had a wide dip (Table 2).

**Table 2:** Distribution of depth with the shape of the notch at 4 kHz in the left ear (n= 14).

Depth (dB)	Shape			Total n (%)
	Normal n (%)	Narrow n (%)	Wide n (%)	
0-25	2 (14.28)	-	-	2 (14.28)
26-50	-	5 (35.71)	5 (35.71)	10 (71.42)
>50	-	-	2 (14.28)	2 (14.28)
Total n (%)	2 (14.28)	5 (35.71)	7 (50)	14 (100)

In the right ear, 6 (42.85%) had a narrow dip and 7 (50%) had a wide dip. Additionally, most of the narrow and wide dips of 4 kHz i.e. 12 (85.71%) cases had depths in between 26 to 50 dB (Table 3).

**Table 3:** Distribution of depth with the shape of the notch at 4 kHz in right ears (n= 14).

Depth (dB)	Shape			Total n(%)
	Normal n (%)	Narrow n (%)	Wide n (%)	
0-25	1 (7.14)	-	-	1 (7.14)
26-50	-	6 (42.85)	6 (42.85)	12 (85.71)
>50	-	-	1 (7.14)	1 (7.14)
Total n (%)	1 (7.14)	6 (42.85)	7 (50)	14 (100)

On the left ear, there were 6 (42.85%) cases of mild hearing loss, in which 3 (21.42%) had narrow and 3 (21.42%) others had wide shaped notches at 4 kHz (Table 4).

**Table 4:** Distribution of shape of the notch with WHO severity in left ears (n= 14).

Shape	Severity (dB)			Total n (%)
	Normal (0-25) n (%)	Mild (26-40) n (%)	Moderate (41-60) n (%)	
Normal	2 (14.28)	-	-	2 (14.28)
Narrow	2 (14.28)	3 (21.42)	-	5 (35.71)
Wide	3 (21.42)	3 (21.42)	1 (7.14)	7 (50)
Total n (%)	7 (50)	6 (42.85)	1 (7.14)	14 (100)

There were 8 (57.14 %) cases of mild hearing loss in the right ears, but a decrease to 4 kHz beyond 25 dB was observed in 13 (92.85 %) cases (Table 5).

**Table 5:** Distribution of shape of the notch with WHO severity in the right ear (n= 14).

Shape	Severity (dB)		Total n (%)
	Normal	Mild	
	(0-25) n (%)	(26-40) n (%)	
Normal	1 (7.14)	-	1 (7.14)
Narrow	5 (35.71)	1 (7.14)	6 (42.85)
Wide	-	7 (50)	7 (50)
Total n (%)	6 (42.85)	8 (57.14)	14 (100)

## DISCUSSION

In our study, the prevalence of NIHL was found to be 2.02% out of 690 patients. In a study done in a tertiary care hospital in Jharkhand, its prevalence in an Outpatient Department setting was similar to our study at 1.83%.^3^ Looking at an occupational noise-induced hearing loss, where prevalence is calculated among high-risk populations, the prevalence is found to be substantially higher, ranging from 26.80-66.4%.^10-12^

Similarly, the ratio of males to females affected with NIHL was around 1.8:1. A study from China also indicates significant sex differences where males are at 2.49 times the higher risk than females.^13^ Meanwhile, in a study of medical students in Pakistan, women were three times more affected by high-frequency sounds indicating NIHL.^14^ Our patients presented with chief complaints of muffled hearing, tinnitus, and vertigo. 13 (92.85%) experienced hearing loss. In a study conducted in a hospital setting in Northern India, 54% complained solely of hearing loss, while the other half complained of tinnitus and hearing loss.^15^ In the same study, NIHL binaural involvement was observed in 74%, which is similar to our study, 78.57%. In a police survey, only 61.64% had a binaural association, which is lower than our study.^12^ The symmetrical binaural mild to moderate hearing loss in NIHL is something that traditional education educates us about. However, a sizable proportion of patients have asymmetrical thresholds.

In our study, roughly half of the cases, 42.90% in the left ear and 57.10% in the right ear had mild hearing loss, while the rest were normal. It is interesting to note that there were only 8 (57.14%) cases of mild hearing loss in the right ears, but a decrease to 4 kHz beyond 25 dB was observed in 13 (92.85%) cases. This suggests that there were more narrow troughs at 4 kHz, averaging close to a lower range of hearing loss. In a separate study of police officers, it was discovered that 93% of them had mild NIHL, 3.5% had moderate NIHL, and 3.5% had severe NIHL.^16^ In a study of Pokhara woodworkers, 30.68% of carpenters were affected, with 14% having mild hearing loss, 14% having moderate hearing loss, and the remaining 3% having severe hearing loss.^11^ In the same research, 44% of sawyers were affected, with 11% having mild hearing loss, 28% having moderate hearing loss, and 5% having severe hearing loss.

In our study, most cases- 71.42% in left ears and 85.71 % in right ears had a depth of 26-50 dB at 4 kHz with as many narrow as wide notch in both ears. A study reported that 28% of the narrow notches and 21% of the wide notches were between 16 and 30 dB, which is lower than our study's findings.^5^

The limitations of this study could be the sample size bias. This single-centre study of patients in various professions cannot be generalized to highrisk occupational groups or the general population. Furthermore, since this is a descriptive study, we were unable to find associations between the variables and disease processes.

## CONCLUSIONS

This study found that the prevalence of NIHL amongst patients requiring PTA evaluation was similar to other studies conducted in similar settings. Although most patients had a hearing degree at a normal range according to WHO standards, the NIHL problem still exists as indicated by the narrow audiometric notch at 4 kHz dip. Given that this is the most prevalent preventable cause of hearing loss, appropriate actions should be taken timely to avoid it.
